# Pregnancy, pregnancy outcomes, and infant growth and development after recovery from Ebola virus disease in Liberia: an observational cohort study

**DOI:** 10.1016/S2214-109X(23)00210-3

**Published:** 2023-07

**Authors:** Mosoka P Fallah, Cavan Reilly, Collin Van Ryn, Moses Badio, Sia Wata Camanor, Stephen G Kaler, Billy Johnson, Romeo Orone, Hilary Flumo, Soka J Moses, Kumblytee L Johnson, Nowiah Gorpudolo, Dehkontee Gayedyu-Dennis, Bonnie Dighero-Kemp, John Fayiah, Lindsay Marron, Lisa E Hensley, Robert J Taylor, Elizabeth S Higgs, H Clifford Lane, James D Neaton, Michael C Sneller

**Affiliations:** Liberia Ministry of Health, Monrovia, Liberia; Division of Biostatistics, University of Minnesota, Minneapolis, MN, USA; Division of Biostatistics, University of Minnesota, Minneapolis, MN, USA; Liberia Ministry of Health, Monrovia, Liberia; John F Kennedy Medical Center, Monrovia, Liberia; Section on Translational Neuroscience, Molecular Medicine Branch, Eunice Kennedy Shriver National Institute of Child Health and Human Development, Bethesda, MD, USA; Center for Gene Therapy, Nationwide Children’s Hospital and Department of Pediatrics, The Ohio State University College of Medicine, Columbus, OH, USA; John F Kennedy Medical Center, Monrovia, Liberia; ELWA Hospital, Monrovia, Liberia; John F Kennedy Medical Center, Monrovia, Liberia; John F Kennedy Medical Center, Monrovia, Liberia; C H Rennie Hospital, Kakata, Liberia; Liberia Ministry of Health, Monrovia, Liberia; Duport Road Clinic, Paynesville, Liberia; Integrated Research Facility, National Institute of Allergy and Infectious Diseases, Fort Detrick, Frederick, MD, USA; Integrated Research Facility, National Institute of Allergy and Infectious Diseases, Fort Detrick, Frederick, MD, USA; Integrated Research Facility, National Institute of Allergy and Infectious Diseases, Fort Detrick, Frederick, MD, USA; Integrated Research Facility, National Institute of Allergy and Infectious Diseases, Fort Detrick, Frederick, MD, USA; National Institute of Allergy and Infectious Diseases, National Institutes of Health, Bethesda, MD, USA; National Institute of Allergy and Infectious Diseases, National Institutes of Health, Bethesda, MD, USA; National Institute of Allergy and Infectious Diseases, National Institutes of Health, Bethesda, MD, USA; Division of Biostatistics, University of Minnesota, Minneapolis, MN, USA; National Institute of Allergy and Infectious Diseases, National Institutes of Health, Bethesda, MD, USA

## Abstract

**Background:**

Minimal data exist on pregnancy following recovery from Ebola in people of child-bearing potential (females aged roughly 18–45 years). The aim of this study was to assess viral persistence or reactivation in pregnancy, the frequency of placental transfer of anti-Ebola IgG antibodies, and pregnancy outcomes in this population.

**Methods:**

In this observational cohort study, we studied self-reported pregnancies in two groups: seropositive people who had recovered from Ebola virus disease (seropositive group) and seronegative people who had close contact with people with Ebola (seronegative group). Participants had enrolled in the PREVAIL III longitudinal study and were exposed during the 2014–2016 Liberian Ebola outbreak. The primary outcome was pregnancy result. We assessed rates of livebirths and other pregnancy results in both study groups, and presence of Ebola RNA by PCR in samples of placenta, maternal and cord blood, breastmilk, and vaginal secretions from people who had recovered from Ebola who conceived a median of 14 months after acute Ebola virus disease. Mixed-model logistic regression evaluated associations between first-reported pregnancy outcome, age, and study group. Growth and neurodevelopment in the infants born to people in the seropositive group were assessed at 6-month intervals for 2 years. Data were accrued by PREVAIL III study staff.

**Findings:**

1566 participants were enrolled between June 17, 2015, and Dec 14, 2017, of whom 639 became pregnant (215 seropositive, 424 seronegative) and 589 reported pregnancy outcomes (206 seropositive, 383 seronegative). 105 infants born to 98 mothers in the seropositive group were enrolled in the birth cohort. Ebola RNA was not detected in 205 samples of placenta, cord blood, or maternal blood taken at birth from 54 mothers in the seropositive group, nor in 367 vaginal swabs. Viral RNA was found in two of 354 longitudinal breastmilk samples. All but one of 57 infants born during these 54 births were seropositive for anti-Ebola antibodies. Neonates showed high concentrations of anti-Ebola IgG, which declined after 6 months. Odds of adverse pregnancy outcome among the two groups were indistinguishable (OR 1·13, 95% CI 0·71–1·79). Compared with WHO standards, infants born to those in the seropositive group had lower median weight and length, and larger median head circumference over 2 years. Compared with a cohort from the USA accrual of gross motor developmental milestones was similar, whereas attainment of pincer grasp and early vocalisation were mildly delayed.

**Interpretation:**

The risks of Ebola virus reactivation in the peripartum and postpartum period and of adverse birth outcomes are low in those who have recovered from Ebola virus disease and become pregnant approximately 1 year after acute Ebola virus disease. The implication for clinical practice is that care of people who are pregnant and who have recovered from Ebola can be offered without risks to health-care providers or stigmatisation of the mothers and their offspring. The implication for prospective mothers is that safe pregnancies are entirely possible after recovery from Ebola.

## Introduction

The outbreak of Ebola virus disease in west Africa between 2014 and 2016 was the largest observed to date. Liberia, Sierra Leone, and Guinea had 28 616 reported cases and 11 310 reported deaths,^1^ although these numbers could be a substantial undercount. The epidemic resulted in many individuals who contracted Ebola virus disease but did not die.^2^ Ebola virus RNA can persist in recovered individuals in immune-privileged sites such as the eyes and brain,^3^ semen,^4,5^ and breastmilk and vaginal secretions.^6^ Viral RNA has been detected as long as 531 days after recovery in semen^7^ and 500 days (due to apparent reactivation during pregnancy) in breastmilk;^8^ detection is intermittent, however, and serial samples from a recovered individual can be negative for weeks or months before subsequently testing positive again. Reported cases of reactivation, transmission, and sequencing information show that persistent virus can cause new resurgent outbreaks up to 7 years after the end of a previous outbreak.^7,9,10^

A 2020^6^ review found that, of 267 pregnancies in which a woman became infected with acute Ebola while pregnant, only 31 ended in livebirths, and only three neonates survived beyond 19 days.^6^ Although the mechanisms behind this negative effect are uncertain, Ebola virus RNA has been found in fetuses after miscarriage and in and stillborn children, and viral RNA has been shown to persist in breastmilk for up to 26 days after symptom onset, and in amniotic fluid for up to 32 days after clearance of the virus from blood.^6^ Infection of health-care workers attending deliveries or miscarriages of women with active Ebola virus disease has also been observed.^11,12^

Little is known about risks associated with pregnancies conceived after recovery from Ebola virus disease. Although a possible instance of viral reactivation during the perinatal period in a pregnant person who had recovered from Ebola virus disease was identified,^9^ neither vertical transmission nor transmission to healthcare workers associated with pregnancy conceived after recovery from Ebola virus disease has been documented. A cohort study^13^ in Liberia found that 19 (28%) of 68 pregnancies in women who had recovered from Ebola ended in miscarriage or stillbirth. The study also found that shorter times between Ebola virus disease recovery and conception were associated with an elevated risk of stillbirth but not higher risk of miscarriage. A cross-sectional, questionnaire-based study^14^ also set in Liberia found that 13 (57%) of 23 of pregnancies in those who recovered from Ebola virus disease ended in stillbirth or miscarriage.

Anecdotal reports during the Liberian Ebola virus disease outbreak also suggested a high rate of miscarriage and other adverse outcomes among recovered individuals who became pregnant, as well as fear among health-care workers and stigmatisation of recovered pregnant people.^12,13,15,16^ To formally evaluate these issues, we launched a study of pregnant women who had recovered from Ebola virus disease and their infants. The primary objectives were to test for viral persistence or reactivation in the peripartum period, identify the frequency of placental transfer of anti-Ebola IgG antibodies, seek evidence of vertical transmission of Ebola virus, and evaluate pregnancy outcomes.

## Methods

### Study design

In this observational cohort study, the birth cohort represents a subgroup within PREVAIL III, a large longitudinal study of people who recovered from Ebola in Liberia that began on June 17, 2015.^4^ Details of enrolment, setting, close-contact identification, baseline examinations, and serology testing have been described previously.^4^ The protocol, informed consent forms, and participant information materials were approved by the institutional review board at the National Institute of Allergy and Infectious Diseases, National Institutes of Health, and the Liberian National Research Ethics Board. Inclusion criteria for PREVAIL III included a diagnosis of Ebola virus disease within 2 years of study launch confirmed by the Liberia Ministry of Health Registry of Ebola virus disease survivors or to have been a close contact of a person with an Ebola virus disease diagnosis, willingness to participate at one of the included health facilities, and informed written consent or assent.

### Participants

Individuals who had recovered from Ebola virus disease and their close contacts were enrolled in PREVAIL III. Participants from both groups who were females aged 18–45 years were identified by physicians at time of enrolment. The group for individuals who had recovered from Ebola virus disease had seropositivity as an inclusion criteria. Conversely, the close contact group were required to be seronegative. During PREVAIL III follow-up visits, which occurred every 6 months, any participant who became pregnant was asked to join the birth cohort study and to enrol their infant upon birth. Recovered individuals from PREVAIL III from the Monsterrado and Margibi counties in Liberia who had a pregnancy ending 37 weeks or less before the substudy start date were asked to enrol their infant retroactively. Recovered individuals who participated in the birth cohort substudy provided informed written consent to collect peripheral blood, vaginal swabs, breastmilk, and cord blood and placenta samples at birth. Recovered individuals also consented to enrol their infants to prospective monitoring of growth and neurodevelopment, and to collection of blood samples from the infant during follow-up exams. No additional consent was required from PREVAIL III participants who had enrolled as close contacts.

### Procedures

Trained trackers maintained contact with the seropositive group, using a tracking and follow-up approach developed for PREVAIL clinical trials.^17^ Trackers helped the group to attend at least four antenatal visits, and made at least two visits to their homes in the first 7 months of pregnancy, and weekly visits thereafter. Regular telephone calls were made to the seropositive group throughout pregnancy, and an emergency contact number was provided. Seropositive participants were instructed to alert the study coordinator at onset of labour so that an ambulance could be dispatched from Refuge Place International, a local non-governmental organisation. Each facility was staffed by an obstetrician or gynaecologist and two midwives who had been trained to collect placenta and cord blood samples at birth, peripheral blood from mothers, and complete case report forms. Delivery sites were supplied with appropriate personal protective equipment.

For the seropositive group, venous ethylenediaminetetraacetic acid blood samples were collected at the time of PREVAIL III enrolment. At delivery, maternal venous blood, umbilical cord blood, a placental swab, and placental tissues were collected. All samples were transported and stored at 4°C and delivered to the testing laboratory within 24 h of collection.

Maternal and fetal surfaces of the placenta were swiped with swabs supplied in universal viral transport system kits (Becton Dickinson, Franklin Lakes, NJ, USA), then placed in vials containing transport medium. Full-thickness 2 cm × 2 cm placenta specimens were obtained and transported in sterile vials; 1 g was processed as a 10% tissue homogenate by grinding into Dulbecco’s Modified Eagle Medium (Gibco, Gaithersburg, MD, USA) using a Fisher Disposable Tissue Grinder (Fisher Scientific, Waltham, MA, USA). Homogenates were clarified by centrifugation at 1800×g for 10 min at 4°C and the supernatant decanted for testing. Maternal and umbilical cord blood samples were centrifuged at 2000×g for 10 min at 4°C, and the plasma decanted. Breastmilk and vaginal swabs were collected 2–4 days after birth, at approximately 2 weeks postpartum, and at 4–6 week intervals for approximately 3 months.

Whole-blood samples were tested for Ebola virus RNA using a real-time reverse transcription PCR-based Cepheid Xpert Ebola Assay (Cepheid, Sunnyvale, CA, USA) according to the manufacturer’s instructions.^18^ Samples were inactivated by adding a 100 μL aliquot of the sample medium to 2·5 mL GeneXpert Lysis Reagent (Guanidinium Thiocyanate) and incubated for 10 min at room temperature; 1 mL of this solution was then added to an Xpert Ebola cartridge and tested within 30 min. The suitability of the Xpert assay for testing placental tissue, vaginal swabs, and breastmilk was evaluated using commercially procured samples spiked with decreasing concentrations of virus. Positive results were confirmed using the Ebola Zaire (EZ1) and Minor Groove Binder (MGB) assays developed by the US Department of Defense.^19,20^ Samples with an invalid test result returned by the Xpert system were retested using the EZ-1 and MGB assays and those results reported.

Anti-Ebola virus IgG was quantified using the Filovirus Animal Nonclinical Group enzyme-linked immunosorbent assay (known as FANG ELISA), as previously described.^4,21^ Umbilical cord blood samples were tested for neonates, and venous blood for infants and adults. Samples with 548 EU/mL or more were categorised as seropositive.

### Outcomes

Outcome measures were rates of livebirths and other pregnancy results; presence of Ebola viral antibodies in maternal and infant serum; presence of Ebola viral RNA in blood, placenta, vaginal secretions, and breastmilk; and infant somatic growth and neurodevelopment. Infants in the seropositive group were evaluated by a paediatrician in a health-care facility at birth and at approximately 6-month intervals thereafter until age 2 years. Infant growth (ie, weight, length, head circumference, and mid-arm circumference) was measured and neurodevelopmental milestones in gross motor, fine motor, and language domains were assessed based in part on the Denver II Developmental Screening Test.^22^

### Statistical analysis

Mixed-model logistic regression was used to test for association between first-reported pregnancy outcome (livebirth *vs* adverse outcome) and the following factors in the pregnant participants: age at the time of pregnancy outcome, BMI at PREVAIL III enrolment, PREVAIL III enrolment site, education level at PREVAIL III enrolment, history of intrauterine fetal demise at PREVAIL III enrolment, PREVAIL III enrolment status (seropositive group *vs* seronegative group), time from Ebola virus disease symptom onset to conception (seropositive group only), and pregnancy end date relative to the start of the birth cohort study (seropositive group only). All models were adjusted for age, BMI, enrolment site, education level, and history of intrauterine fetal demise. Generalised estimating equations were used to adjust for repeated measurements (some first-reported pregnancies were multiple pregnancies ie, twins or higher multiples). An adverse outcome was defined as a stillbirth at 20 weeks or more of pregnancy, miscarriage before 20 weeks of pregnancy, ectopic pregnancy, or livebirth with subsequent neonatal death (within 28 days). Missing outcome data was multiply imputed using the mice package in R and the pregnancy outcome analyses were repeated in the combined seropositive and seronegative cohort. The models fit with imputed data did not adjust for the small number of repeated measurements. Further sensitivity analyses were done, in which all missing outcomes among the seropositive group were categorised as adverse outcomes and all missing outcomes among the seronegative group were categorised as livebirths, and vice versa, to assess the effect of these extreme cases on results.

Weight, length, head circumference, and mid-arm circumference of birth cohort infants were compared with WHO growth standards.^23^ Model-estimated percentiles were plotted against WHO percentiles. The lqmm package in R was used to fit quantile regression models, which regressed each growth measure against Box-Cox transformed age in months. R (version 3.5) was used for statistical analysis.

### Role of the funding source

Individuals employed by the funders of the study (National Institute of Allergy and Infectious Diseases or the Liberia Ministry of Health) participated in study design, data collection, data analysis, data interpretation, and writing of the report. The Ministry of Health Liberia had no role in study design, data collection, data analysis, data interpretation, or writing of the report.

## Results

Between June 18, 2015, and Dec 14, 2016, 1566 adults and 105 infants born between Jan 8, 2016, and Dec 6, 2017, were allocated to the birth cohort (figure). The ethnicity of all participants was Black. During the study, 215 (45·9%) of 468 people in the seropositive group became pregnant at least once, compared with 424 (38·6%) of 1098 in the seronegative group. Median follow-up time was 4·1 years (IQR 3·1–4·5) for the seropositive group and 3·5 years (2·9–4·0) for the seronegative group. All individuals in the seropositive group attended at least four antenatal visits. Post-delivery, 98 birthing parents enrolled 105 infants in the substudy for follow-up, including three sets of twins from first pregnancies after enrolment and four non-twin siblings from second pregnancies after enrolment. We obtained maternal and infant samples at the births of 57 infants (including all three sets of twins; figure). The median time from symptom onset to conception of these 54 pregnancies was 14 months (IQR 10–20; table 1).

Of the 205 samples taken at the 54 births, none were positive by PCR for Ebola virus RNA (appendix p 11). The 95% CI for the probability that a person would test positive (54 births and no positive samples) was 0–0·07. None of the providers attending these births were subsequently diagnosed with Ebola virus disease. Of 367 vaginal swabs from 79 participants in the seropositive group, none were positive for Ebola virus RNA. Of 354 breastmilk samples from 86 individuals in the seropositive group, two were positive for Ebola virus RNA (appendix p 11). We tested these specimens multiple times using the Xpert assays and results were confirmed using the EZ1 and MGB assays that targeted distinct regions of the GP and NP genes. Ct values for the NP target in the Xpert assay ranged from 33·9–38·2, and from 36·9–40·1 in the MGB assay. For the GP target, values ranged from 37·5 to not detected in the Xpert assay, and from 37·1 to not detected in EZ1. All subsequent breastmilk samples obtained from the two birthing parents with Ebola virus-positive samples (n=18 and n=15) were negative for viral RNA.

As expected for a seropositive cohort, all 98 birthing parents who enrolled an infant in the birth cohort had stable concentrations of IgG antibodies against Ebola virus surface glycoprotein at birth and follow-up (table 2). The median concentration of antibodies in the neonates was comparable to the birthing parent’s concentrations at birth but decreased during the following 12 months (table 2). All but one of 54 infants tested was seropositive at birth; however, by 6 months of age, 45 were seronegative (ie, <548 EU/mL; table 2; appendix p 1).

158 (75·6%) of 209 known first pregnancy outcomes among the seropositive group were livebirths, while 295 (75·6%) of 390 known pregnancy outcomes among the seronegative group were livebirths (table 3; appendix p 2). The adjusted odds ratio (OR) of an adverse pregnancy outcome in the seropositive group versus the seronegative group was 1·13 (95% CI 0·71–1·79; appendix p 12). Adverse outcomes were not significantly associated with time elapsed from Ebola virus disease admission to estimated date of conception (OR 1·12, 95% CI 0·78–1·61) or with whether the pregnancy ended before or after the substudy began (1·44, 0·55–3·76; appendix p 12). A sensitivity analysis to account for missing data using multiple imputation yielded very similar results (appendix pp 13–14). When all missing outcomes among the seropositive group were categorised as adverse outcomes and all missing outcomes among the seronegative group contacts were categorised as livebirths, the seropositive group had significantly greater odds of an adverse outcome (1·57, 1·01–2·43; appendix p 15). The difference was not significant when missing outcomes were categorised as adverse outcomes for the seronegative group and livebirths for the seropositive group (0·65, 0·42–1·01; appendix p 15). Individuals in the seropositive group who became pregnant after the substudy start date were more likely to give birth in a hospital than those who gave birth beforehand but their rates of adverse outcomes were similar (appendix p 16).

Eight infants born to participants in the seropositive group died between 1 month and 20 months of age, with seven of these deaths before 12 months of age. Causes of death included malaria (two infants), anaemia (two infants), unknown febrile illness (one infant), and acute diarrhoeal illness (one infant). For two infants, the cause of death remains unknown.

The median and first and third quartile values for weight in the first 2 years of life were lower compared with WHO standards (appendix p 3). In female infants, the discrepancy in median weight began at 3 months of age and was approximately 90% of the WHO median at 12 months and 18 months of age while in males the discrepancy began at 1·5 months of age and was approximately 92% of the WHO median at 12 months of age and 95% at 18 months. Median linear growth diverged from WHO standards later (8–10 months of age) and was approximately 98% of the standard by 18 months of age (appendix p 4). By contrast, median head circumference, an accurate reflection of brain growth, was 3–4% higher in participating infants compared with WHO medians; this discrepancy began at 9 months in females and at 14 months in males (appendix p 5). Mid-arm circumference, a measure of general nutritional status, appeared similar to WHO standards (appendix p 6).

The first and third quartile ages at which selected gross motor milestones (eg, independent sitting, pulling to stand, walking, and running) were within the ranges predicted by the Denver II Developmental Screening Test (appendix p 7).^22^ This prediction also held for certain fine motor milestones (eg, reaching for objects and transferring objects), although development of a pincer grasp appeared delayed (first quartile 12 months, third quartile 15 months, appendix p 8) compared with the Denver II range (7–10 months).^24^ In terms of language development, slightly later onset of babbling (first quartile 6 months, third quartile 8 months) and syllable formation (first quartile 12 months, third quartile 14 months) compared with Denver II ranges (4–6 months for babbling and 5–10 months for syllable formation)^24^ were noted; however, early word acquisition appeared similar to the Denver II prediction (10–18 months, appendix p 9).^22^ Personal social development did not differ notably from predictions (appendix p 10).^24^

## Discussion

Our findings suggest that people who become pregnant within 10–20 months (median 14 months) after surviving Ebola are unlikely to have reactivation of persistent virus in the peripartum period and are therefore unlikely to transmit Ebola virus to their infants, contacts, or caregivers. Neonates of people who have recovered from Ebola virus disease had high concentrations of transplacental Ebola antibodies, suggesting robust early protection from Ebola virus disease. The decrease in infant anti-Ebola antibodies after birth is consistent with the expected decay of maternally acquired IgG antibodies observed with other pathogens.^24,25^ The absence of clinical manifestations of Ebola virus disease in the infants indicate that in utero, perinatal, or breastmilk transmission of Ebola virus did not occur in our cohort.

Of all study samples tested for Ebola virus RNA, only two breastmilk samples were positive from two different people and were collected 3 days after birth, at the same site, on the same day. The time from Ebola virus disease symptom onset to birth for these two people was 15 months and 16 months. We tested these specimens multiple times using the Xpert assays and results were confirmed using the EZ1 and MGB assays that targeted distinct regions of the GP and NP genes. All subsequent breastmilk samples obtained from the two birthing parents were negative for viral RNA. The confirmation of positive results on multiple assays is consistent with low presence of viral RNA. Given the unusual coincidence of when and where the samples were obtained, we cannot exclude contamination from an unknown source during specimen collection or processing. However, the detection of EBV RNA using two distinct assays and the amount of time that had passed since the laboratory had handled samples from acute cases strongly support that the results were not the product of accidental contamination. Our data support that the risk of Ebola virus transmission via breastmilk is minimal, especially with the high concentrations of anti-Ebola serologies in infants, and is far outweighed by the benefits of breastfeeding.

The odds of livebirth between those who had recovered from Ebola virus disease and those who had close contact were indistinguishable, indicating that past Ebola virus disease infection does not heighten risk in recovered peoples’ pregnancies. These results did not change meaningfully in sensitivity analyses. Our pregnancy outcome findings differ from those of Godwin and colleagues^14^ who found in a retrospective questionnaire study that only ten (43·4%) pregnancies in their cohort of 23 pregnancies to people who had recovered from Ebola virus disease ended in livebirth, a substantially smaller proportion than the 80·4% of pregnancies we report here. Their study did not report the interval from Ebola virus disease to conception, which might have been shorter, potentially rendering livebirth less likely.^14^ Four of the present authors also previously reported a smaller retrospective analysis^13^ of 68 pregnancies in which adverse outcomes were higher than found in this study, and hypothesised that stillbirths might occur more frequently with a shorter interval from acute Ebola virus disease to conception. Median time from Ebola virus disease to conception in that study was 5 months, versus 14 months in the current study.

Until the present study, few data had been published concerning the growth and development of infants born to people who have had Ebola virus disease.^26,27^ The mortality rate among birth cohort infants younger than 1 year corresponded to 67 per 1000 livebirths; by comparison, the mortality rate in Liberia for children younger than 1 year is 58 per 1000.^28^ Apart from subtle differences in growth, and fine motor and language development, surviving infants in our study did not have significant health problems. The apparent delays in acquisition of some developmental milestones might reflect inaccuracies possible when standards from high-income settings are applied to low-income settings.^29^ The apparently lower rate of weight gain in infancy, especially in female offspring (appendix p 3), merits follow-up with appropriate comparator cohorts. Previous study of children without HIV born to people who are HIV-1 seropositive revealed deficits in linear growth.^30^

There are several limitations to this study. Placental swabs and 1 g samples of placental tissues might not detect virus present elsewhere in the placenta. We took samples from pregnancies conceived a median of 14 months after Ebola virus disease recovery; pregnancies closer to the time of onset of Ebola virus disease recovery might have a greater likelihood of persistent virus and negative outcomes of pregnancy.^13,14^ Specimens were not available for all adverse outcomes, and were only available for one stillbirth and three livebirths with subsequent neonatal death. Samples from adverse pregnancy outcomes, including miscarriage, could contain Ebola virus RNA. Unrecognised pregnancies might have been lost, especially early in gestation, which could have biased the results. Finally, when the birth cohort substudy began, people who had recovered from Ebola virus disease were offered help in accessing antenatal care and birth facilities and those who had close contact were not, which have been shown to reduce risks of maternal and infant mortality.^31^ The sharp rise in the proportion of study survivors who gave birth in medical facilities after the study began suggests that these efforts had an effect (appendix p 16). We do not have data on the number of antenatal visits or delivery site for pregnant people who had close contact and thus cannot determine the extent to which extra care might have improved pregnancy outcomes for those who had recovered from Ebola virus disease.

This study adds substantially to the knowledge about pregnancy in Ebola virus disease survivors. Our findings suggest that people who recover from Ebola virus disease can safely carry a pregnancy especially when conception occurs more than a year after acute Ebola virus disease, although longer term follow-up of growth and development in babies born to these people is needed. Overall, these findings should be reassuring to people who have had Ebola virus disease, their families, and health-care providers since Ebola reactivation in the peripartum period and transmission to infants appears unlikely.

## Supplementary Material

Fallah_Lancet_Glob_Health_2023_supp

## Figures and Tables

**Figure:**
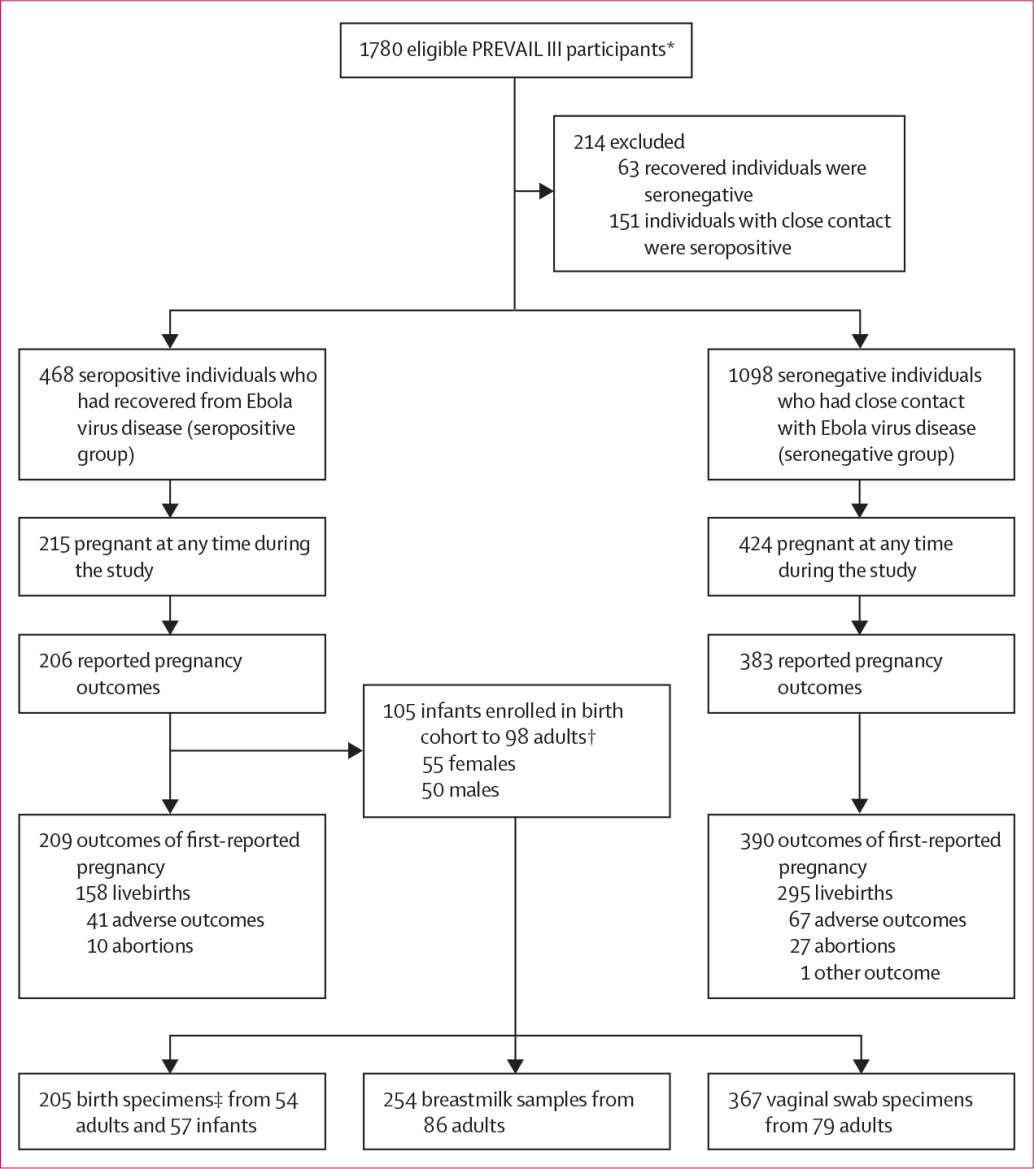
Trial profile *Eligible participants had to be women aged roughly 18–45 years. †Includes three sets of twins from first pregnancies after enrolment and four non-twin siblings from subsequent pregnancies. ‡Includes placental swabs, placental tissue, cord blood, and maternal blood.

**Table 1:** Baseline characteristics

	First-reported-pregnancy outcome analysis			Seropositive group members enrolling an infant (n=98)
	Seropositive group* (n=206)	Seronegative group† (n=383)	Overall (n=589)	

Age at enrolment, years	25 (20–30)	23 (18–28)	24 (19–29)	25 (21–30)
Enrolment site				
John F Kennedy Medical Center	137 (66·5%)	174 (45·4%)	311 (52·8%)	61 (62·2%)
C H Rennie Hospital	27 (13·1%)	122 (31·9%)	149 (25·3%)	17 (17·3%)
Duport Road Clinic	42 (20·4%)	87 (22·7%)	129 (21·9%)	20 (20·4%)
Months from Ebola virus disease symptom onset to enrolment‡	11 (10–12)	17 (14–20)	15 (12–18)	11 (10–13)
Gravidity at PREVAIL III enrolment	2 (1–4)	2 (0–4)	2 (0–4)	2 (1–4)
Parity at PREVAIL III enrolment	1 (0–3)	1 (0–2)	1 (0–3)	2 (1–3)
History of IUFD ≥20 weeks	14 (6·8%)	20 (5·2%)	34 (5·8%)	6 (6·1%)
History of IUFD <20 weeks	49 (23·8%)	53 (13·8%)	102 (17·3%)	27 (27·6%)
History of induced abortion	45 (21·8%)	86 (22·5%)	131 (22·2%)	15 (15·3%)

Data are n (%) or median (IQR). Enrolment refers to enrolment in PREVAIL III. IUFD=intrauterine fetal demise.

* The seropositive group included individuals who had previously recovered from Ebola virus disease and were seropositive.

† The seronegative group included individuals who had close contact with someone with Ebola virus disease and were seronegative.

‡ For the seronegative group this is the time from Ebola virus disease symptom onset in the individual they had contact with.

**Table 2:** Ebola virus antibody concentrations for seropositive mothers and infants enrolled in the birth cohort

	n	Ebola virus antibody concentration, EU/mL	Age, months

**Seropositive adults**			
PREVAIL III enrolment	98	17 740 (11 200–26 510)	··
Birth of first infant enrolled in birth cohort*†	51	16 370 (9296–23 070)	··
6-month study visit	90	15 350 (10 570–22 740)	··
12-month study visit	87	15 560 (10 340–23 280)	··
**Infants**			
Birth cohort enrolment (at birth, cord blood)*	54	15 920 (6693–23 790)	0
Birth cohort enrolment (after birth, blood)	12	171 (90–956)	6 (4–7)
6-month study visit	74	114 (59–283)	7 (6–8)
12-month study visit	11	37 (30–48)	13 (12–16)

Data are n or median (IQR). Birth specimens include cord blood only; blood draws were performed at other timepoints. Antibody levels in infants declined by approximately 1852 EU per month on average.

* Median time from enrolment to delivery of first infant to be enrolled in the birth cohort was 9 months (IQR 5–15).

† No antibody results for three of the 54 seropositive mothers with PCR results from blood samples obtained at the time of birth.

**Table 3:** Outcomes of first reported pregnancies in the seropositive and seronegative groups

	Seropositive group (n=209)*	Seronegative group (n=390)†	Adjusted OR (95% CI) for seropositive vs seronegative group

Livebirth	158 (75·6%)	295 (75·6%)	1·00 (0·67–1·49)
Livebirth with subsequent neonatal death	3 (1·4%)	13 (3·3%)	0·43 (0·14–1·33)
Stillbirth (≥20 weeks)	6 (2·9%)	8 (2·1%)	1·17 (0·37–3·72)
Miscarriage (<20 weeks)	31 (14·8%)	45 (11·5%)	1·46 (0·87–2·45)
Induced abortion	10 (4·8%)	27 (6·9%)	0·69 (0·32–1·49)
Ectopic pregnancy	1 (0·5%)	1 (0·3%)	··
Other‡	0	1 (0·3%)	··

Data are n (%) or OR (95% CI). ORs are adjusted for age at time of pregnancy outcome and PREVAIL III site and were estimated using generalised estimating equations logistic regression models, which accounted for correlatedness arising from repeated measurements (outcomes are reported by fetus, and some pregnancies were multiple pregnancies). OR=odds ratio.

* The seropositive group included individuals who had previously recovered from Ebola virus disease and were seropositive.

† The seronegative group included individuals who had close contact with someone with Ebola virus disease and were seronegative.

‡ One outcome categorised as “Other” was specified as “traumatic abortion <20 weeks’ gestation” by which miscarriage was meant.

## Research in context

### Evidence before this study

On June 17, 2015, the Partnership for Research on Ebola Virus in Liberia (PREVAIL) launched the PREVAIL III study to follow up people who had recovered from Ebola virus disease and their close contacts for up to 5 years. At that time, Ebola virus disease during pregnancy was known to cause fetal loss and anecdotal evidence suggested that pregnancies conceived after recovery from Ebola virus disease might also be at increased risk. In addition, stigmatisation and fear of contracting Ebola virus disease from the pregnant person during delivery by health-care providers were preventing proper antenatal and obstetric care. Little was known about risks to health-care providers from persistent virus; potential for reactivation of Ebola virus in the peripartum period; rates of adverse pregnancy outcomes among those who had recovered; or risks to, and development of, infants born to people who had recovered from the disease.

### Added value of this study

On Dec 24, 2015, we launched the birth cohort substudy within the PREVAIL III longitudinal survival study to address these unknowns. To our knowledge, this study is the largest study of pregnancy in people who have recovered from Ebola virus disease and are seropositive (seropositive group) to date, and the only such study to include seronegative people who have had close contact with these people as a comparison group (seronegative group). We did not detect Ebola virus in any sample from the 54 births at which samples were collected. All but one of 57 infants born during these 54 births were seropositive for anti-Ebola antibodies, with titres decreasing sharply in the first 6 months of life. All 367 vaginal swabs from the seropositive group were negative for viral RNA, while two of 354 maternal breastmilk samples tested positive at a single timepoint. The seropositive group was at approximately the same risk of adverse pregnancy outcomes as the seronegative group contacts, with some important caveats, including that we provided the seropositive group but not the seronegative group help in obtaining antenatal care and transportation to a hospital at birth. Infants born to the seropositive group showed minor differences in somatic growth compared with global standards and subtle delays in attaining pincer grasp and early vocalisation compared with standards derived from a large cohort in the USA.

### Implications of all the available evidence

The absence of Ebola virus in samples taken at birth indicates that seropositive individuals who recover before conception, their infants, and health-care workers attending the births are not at increased risk of Ebola virus disease. Such information should help to alleviate the stigmatisation of people who have recovered from the disease seeking antenatal and obstetrical care. Infants born to the seropositive group passively acquire anti-Ebola IgG during gestation, suggesting they are protected against Ebola virus disease in their first 6 months of life. People who have recovered from Ebola virus disease approximately 1 year before conception and receive adequate antenatal care appear to have similar chances of delivering a healthy infant as individuals who have not had the disease. Longer term follow-up of growth in infants born to those who have recovered from Ebola virus disease with an appropriate comparator cohort is needed.
