# Supplementary material for: Pregnancy, pregnancy outcomes, and infant growth and development after recovery from Ebola virus disease in Liberia: an observational cohort study
Source: Lancet Glob Health. Author manuscript; Available in PMC 2026 May 18. (PMC13181420; doi:10.1016/S2214-109X(23)00210-3)
Supplement: Fallah_Lancet_Glob_Health_2023_supp [file NIHMS2149325-supplement-Fallah_Lancet_Glob_Health_2023_supp.pdf]

THE LANCET
Global Health

Supplementary appendix

This appendix formed part of the original submission and has been peer reviewed. We post it as supplied by the authors.

Supplement to: Fallah MP, Reilly C, Van Ryn C, et al. Pregnancy, pregnancy outcomes, and infant growth and development after recovery from Ebola virus disease in Liberia: an observational cohort study. *Lancet Glob Health* 2023; **11:** e1053–60.

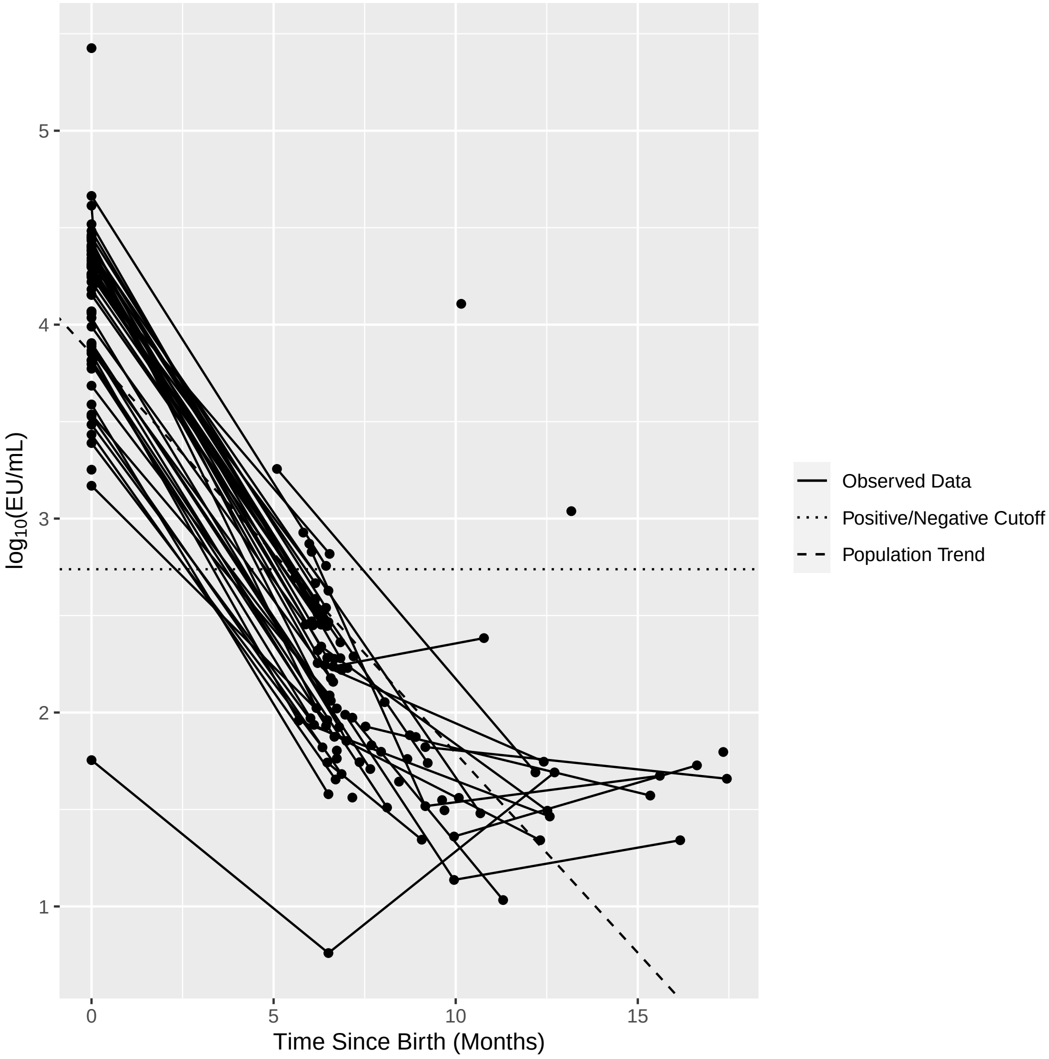

Figure S1: Antibody levels over time for infants enrolled in the birth cohort.

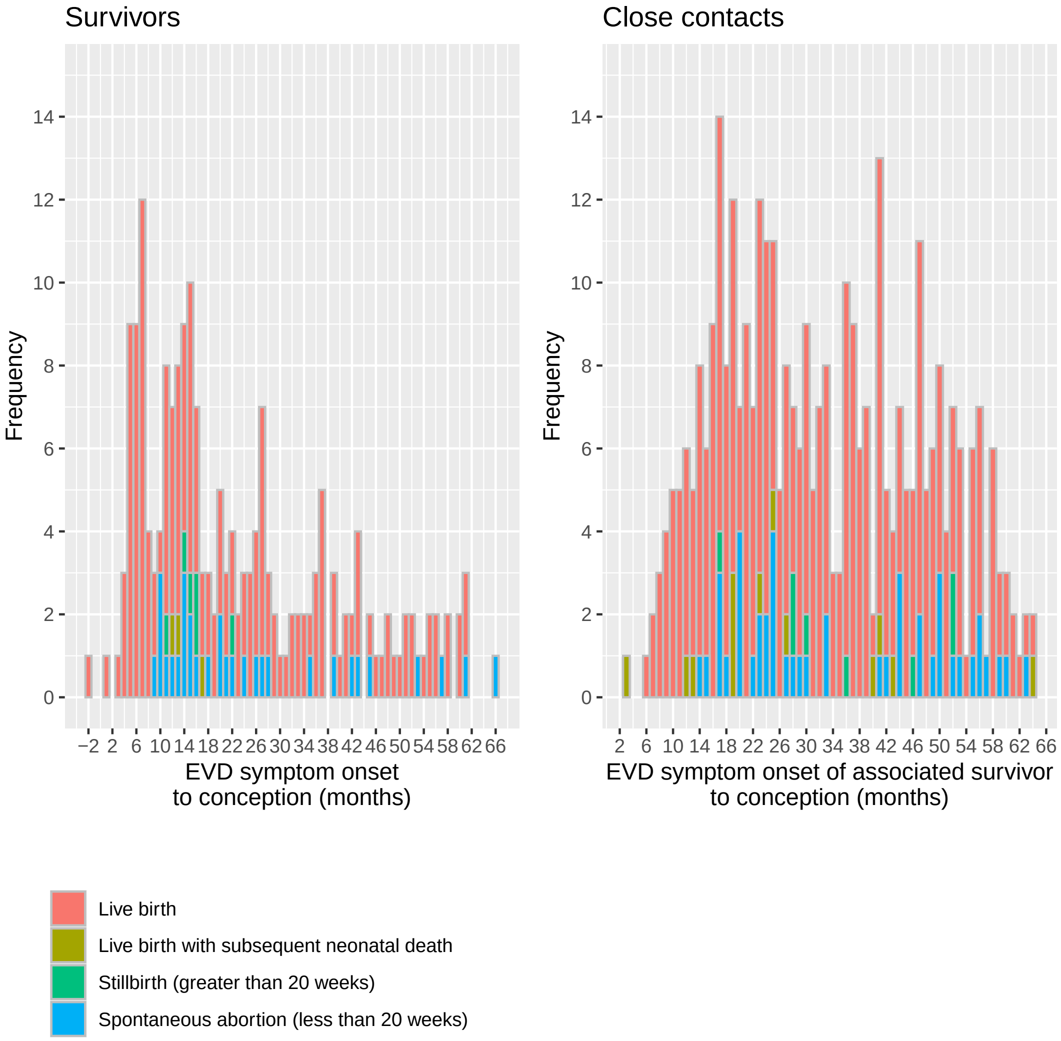

Figure S2: Distribution of first reported pregnancy outcomes by months from EVD symptom onset to conception (for survivors) and months from EVD symptom onset in associated survivors to conception (for close contacts).

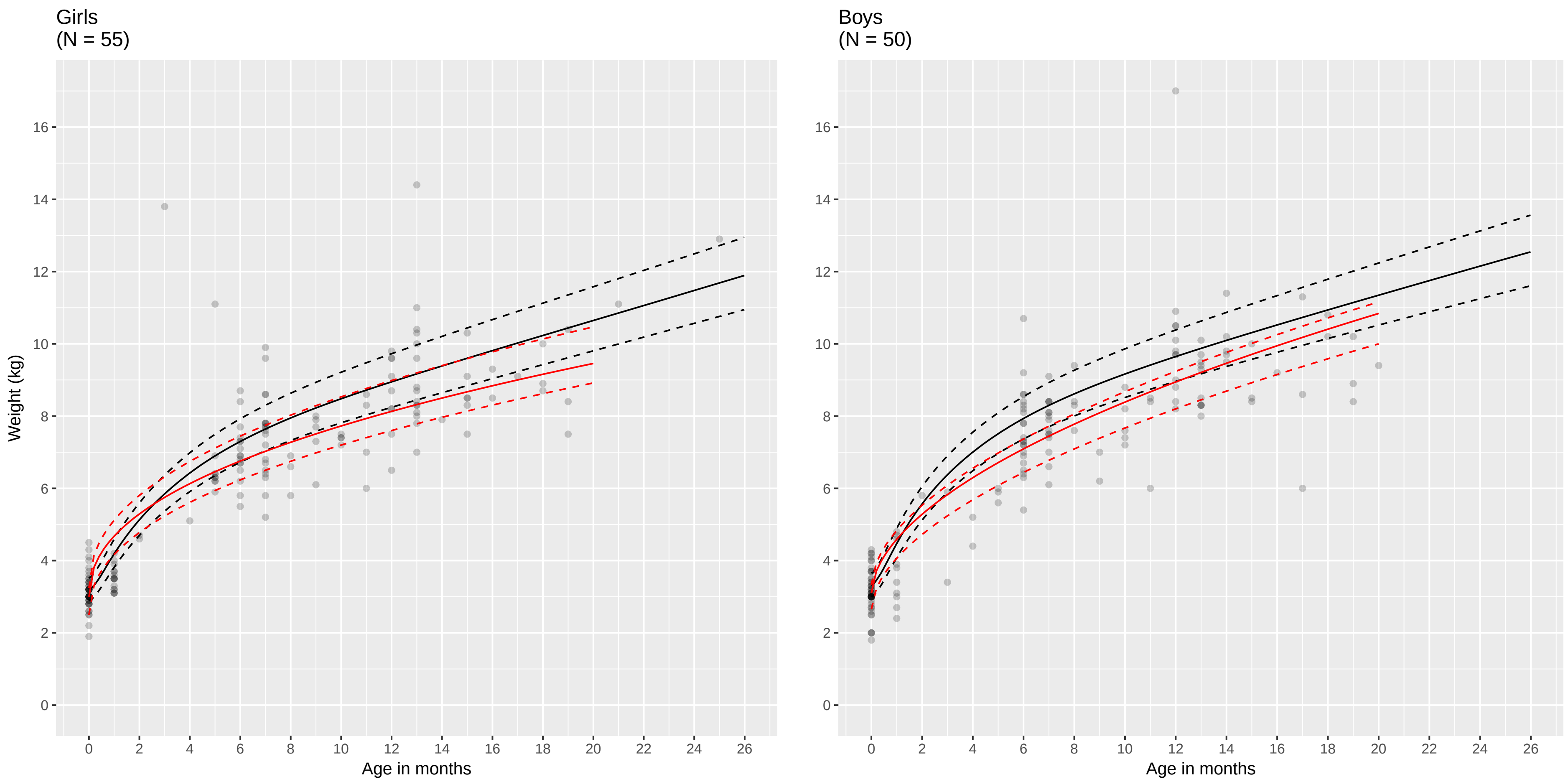

Figure S3: Weight across time for infants enrolled in the birth cohort. The black solid and dashed lines show the median and first and third quartiles of the WHO growth standards. The red lines show the estimated median and first and third quartiles for infants in the birth cohort.

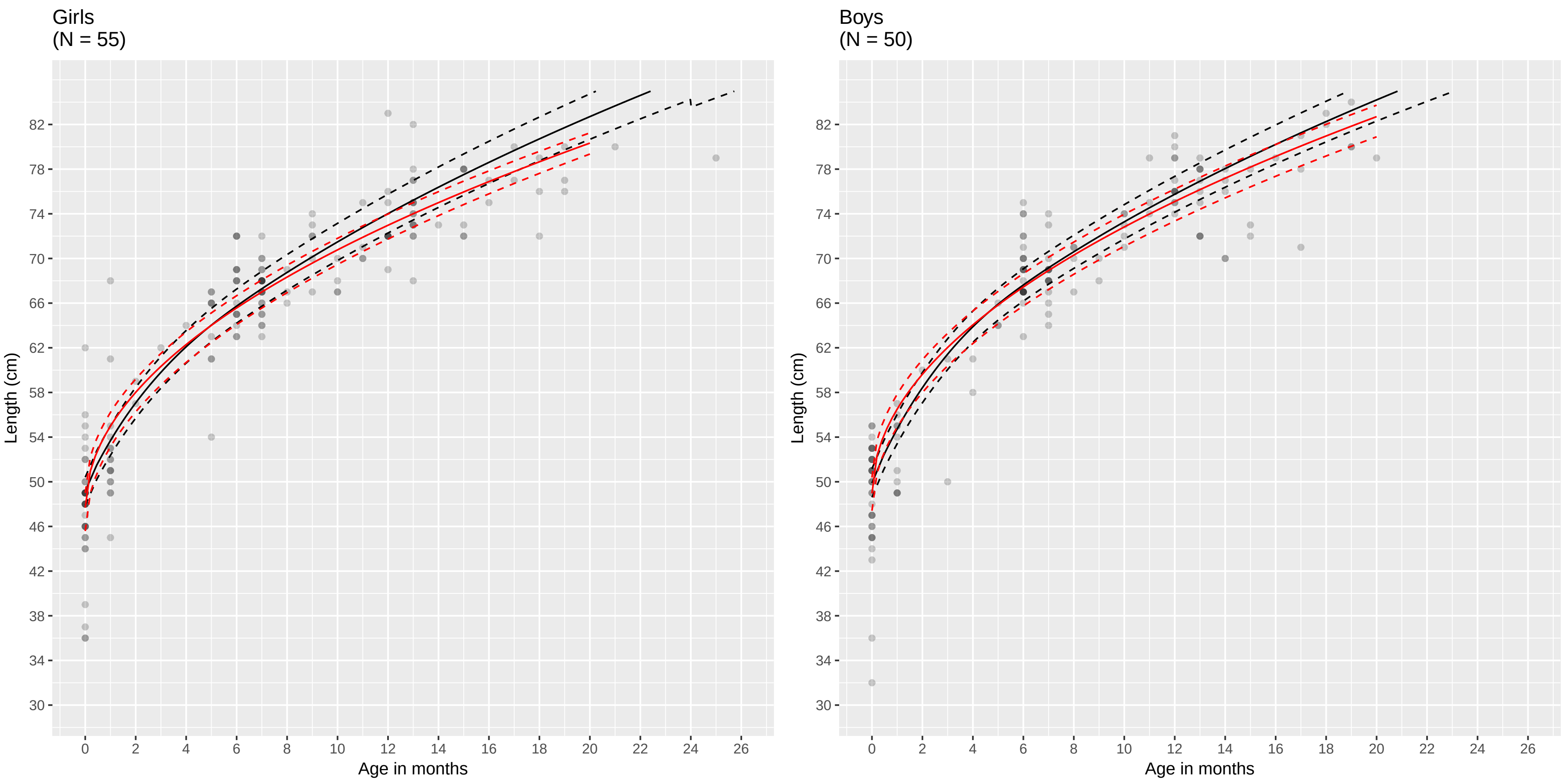

Figure S4: Length across time for infants enrolled in the birth cohort. The black solid and dashed lines show the median and first and third quartiles of the WHO growth standards. The red lines show the estimated median and first and third quartiles for infants in the birth cohort.

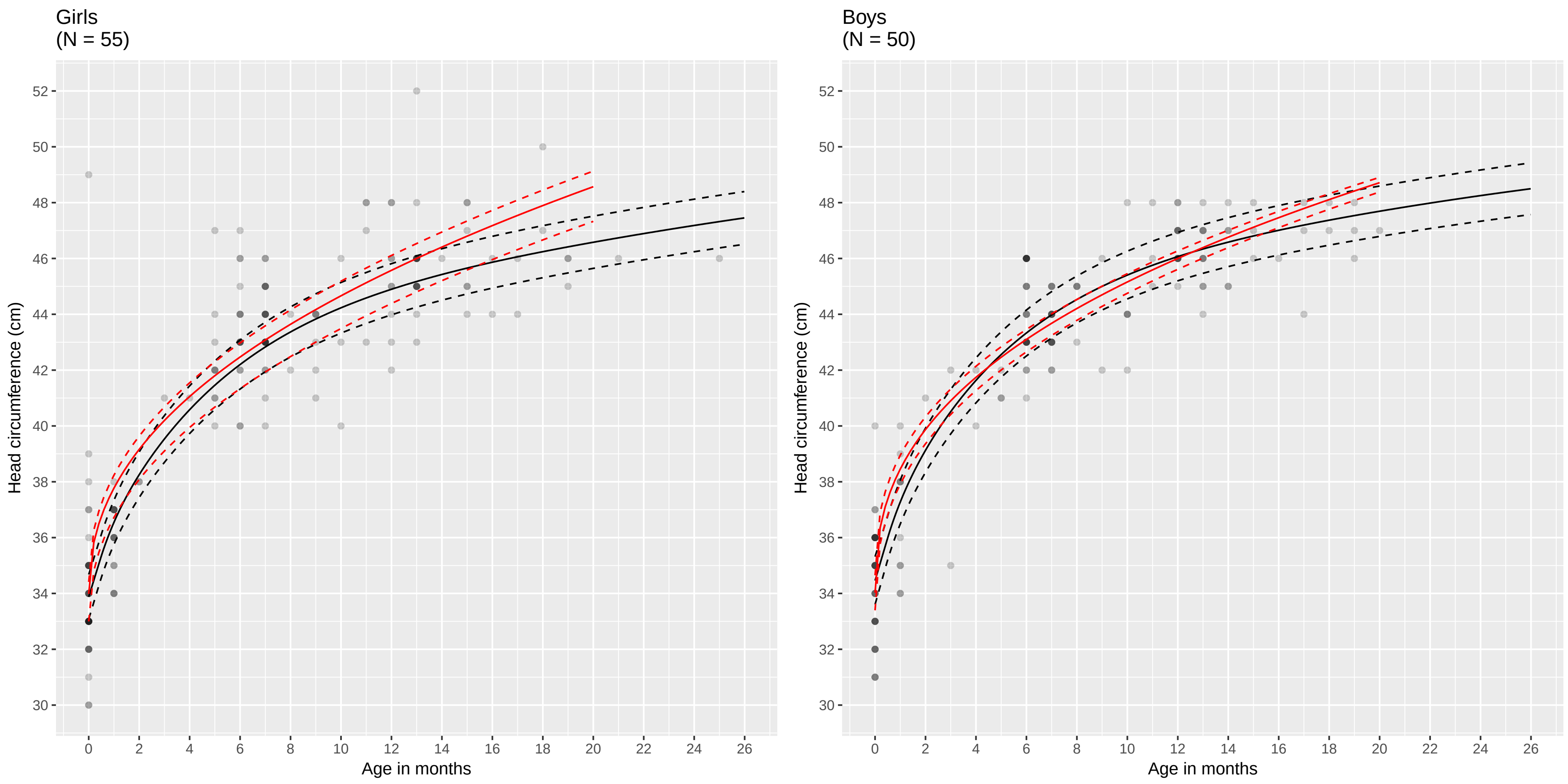

Figure S5: Head circumference across time for infants enrolled in the birth cohort. The black solid and dashed lines show the median and first and third quartiles of the WHO growth standards. The red lines show the estimated median and first and third quartiles for infants in the birth cohort.

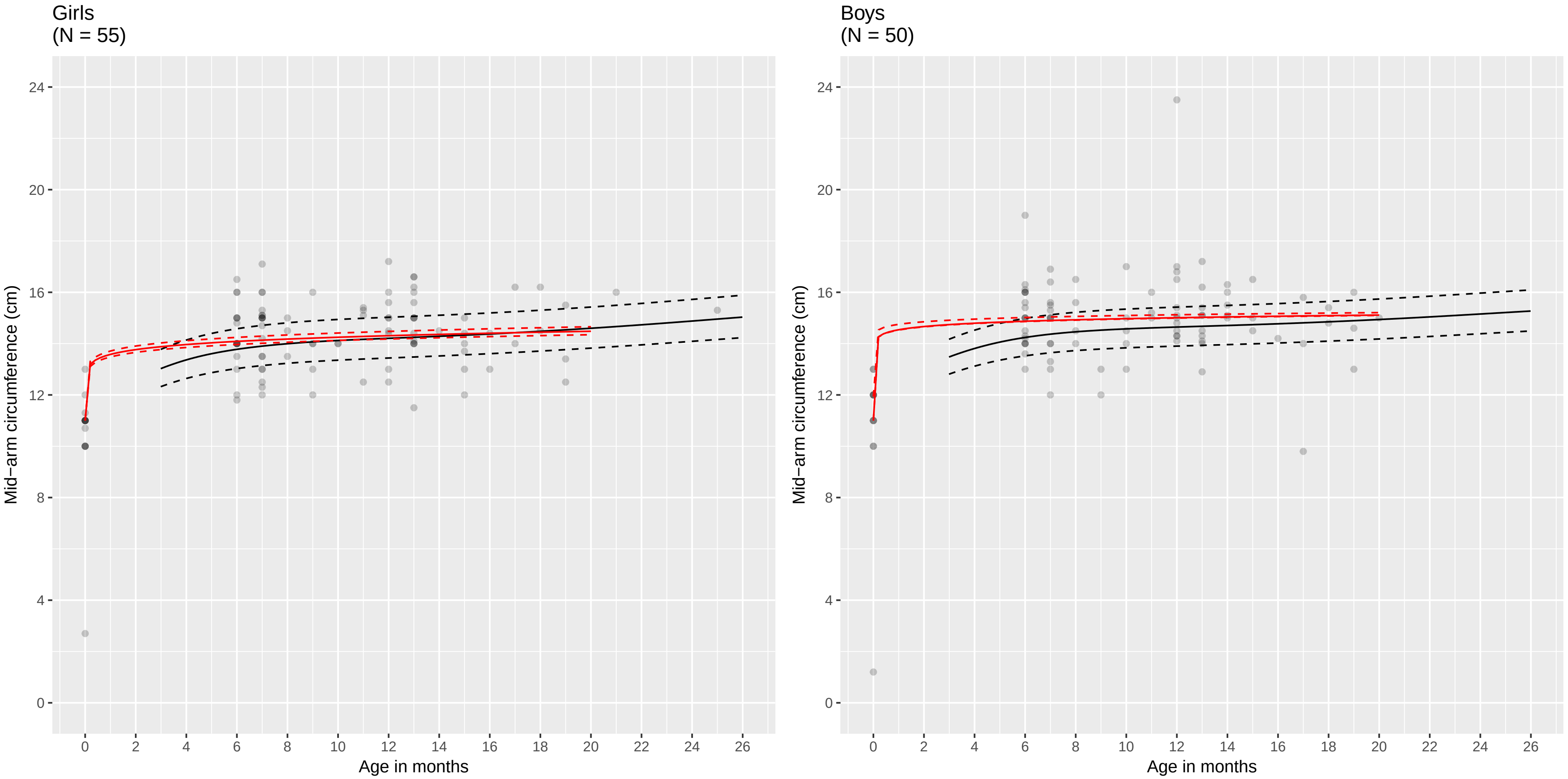

Figure S6: Mid-arm circumference across time for infants enrolled in the birth cohort. The black solid and dashed lines show the median and first and third quartiles of the WHO growth standards. The red lines show the estimated median and first and third quartiles for infants in the birth cohort.

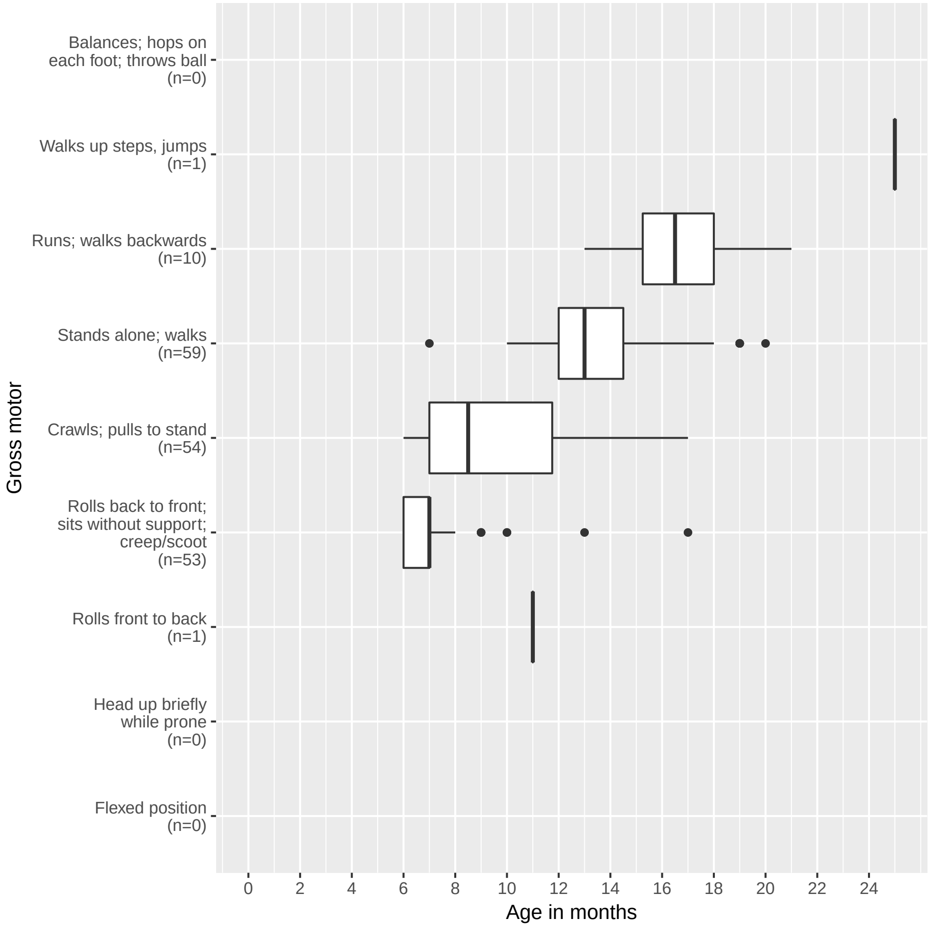

Figure S7: Summary of ages at which gross motordevelopmental milestones where reached for infants in the birth cohort.

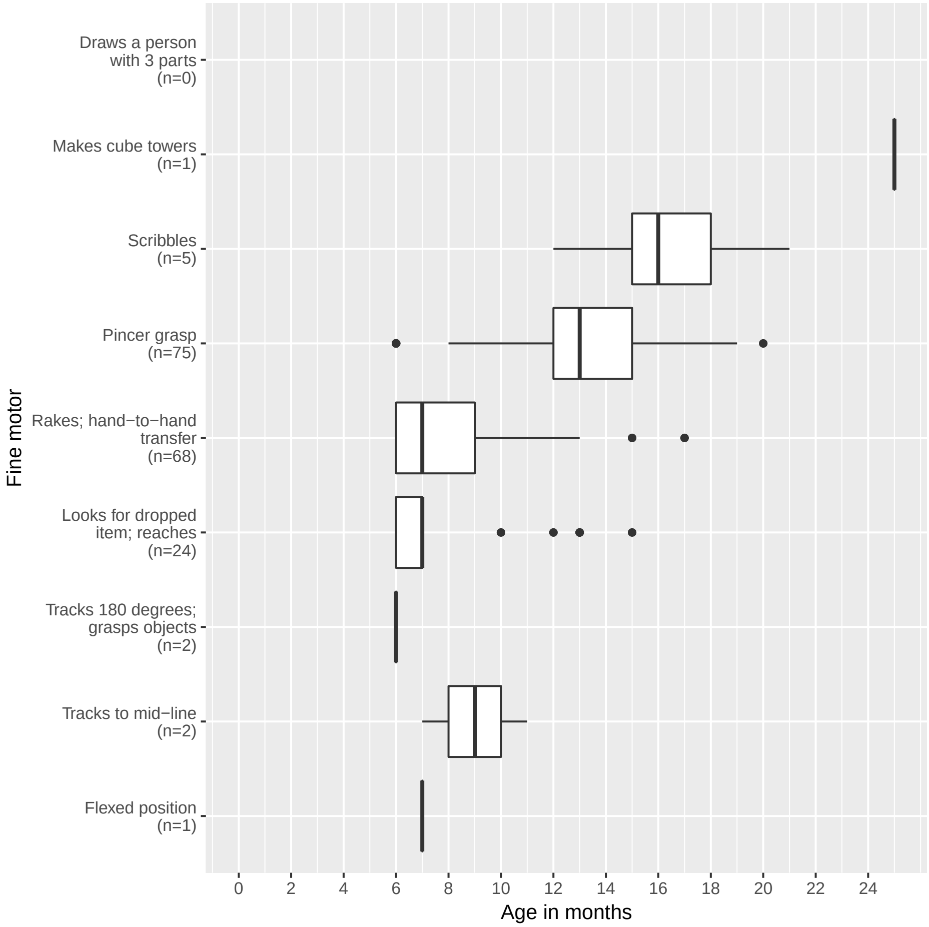

Figure S8: Summary of ages at which fine motordevelopmental milestones where reached for infants in the birth cohort.

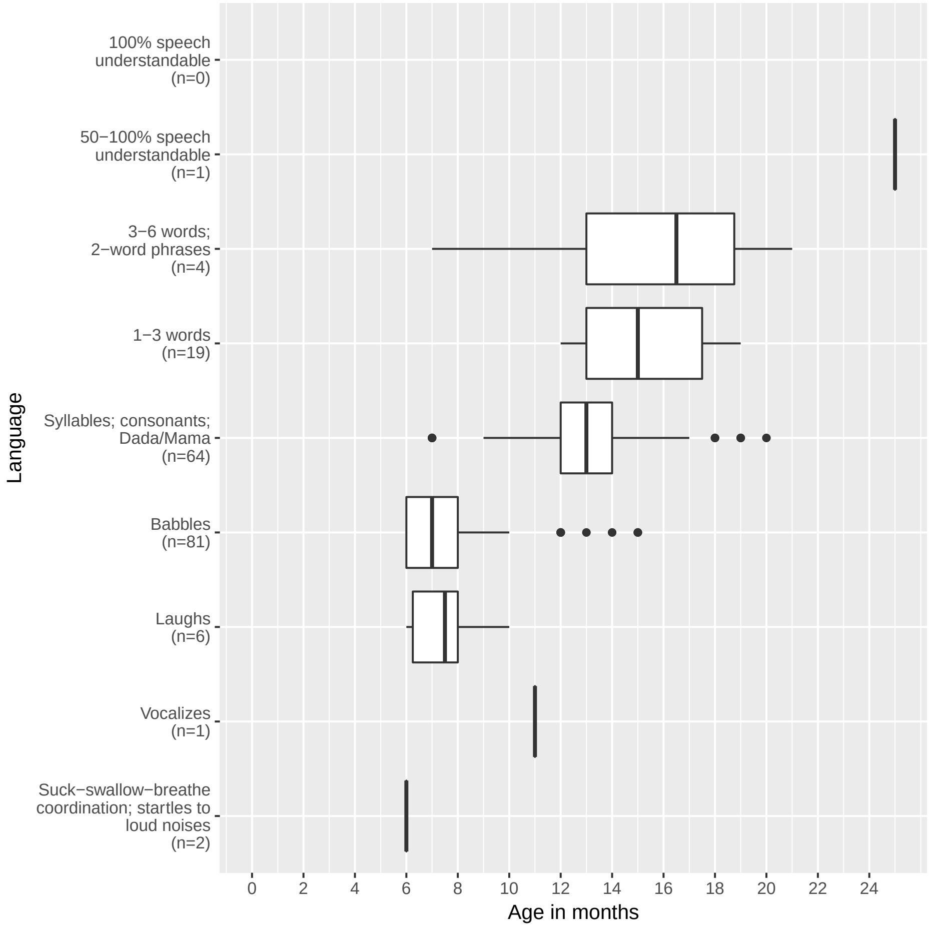

Figure S9: Summary of ages at which linguistic/cognitive neurodevelopmental milestones where reached for infants in the birth cohort.

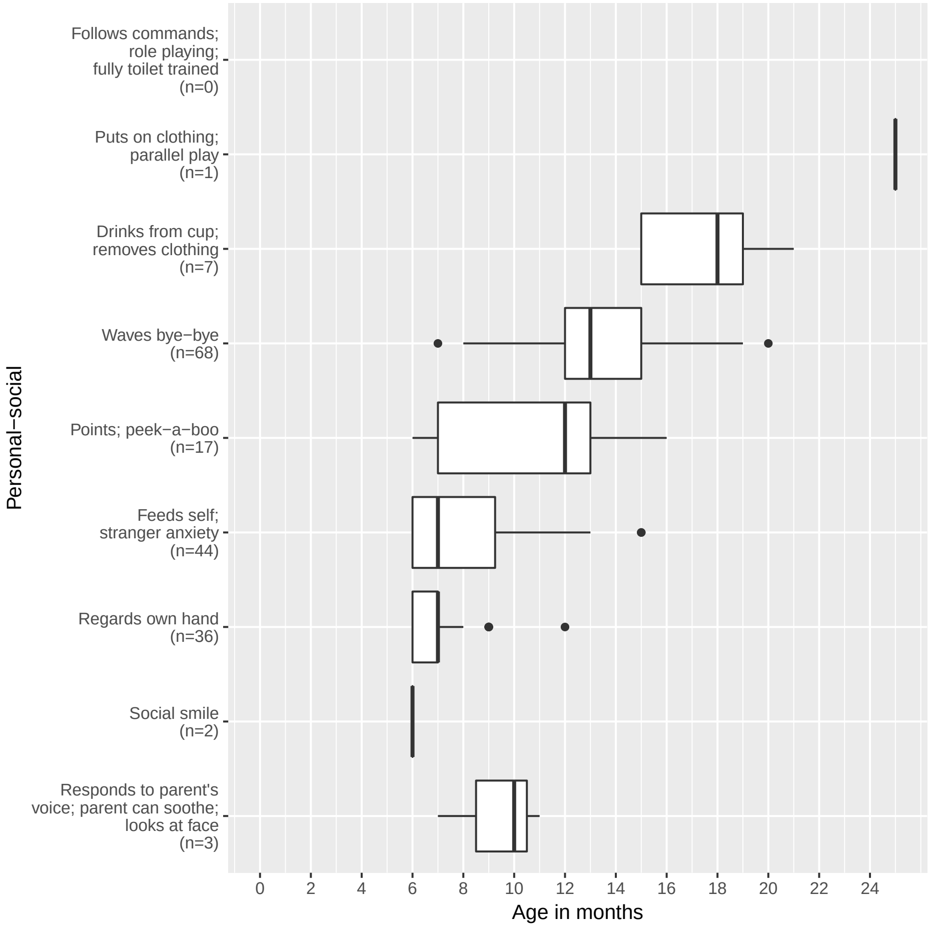

Figure S10: Summary of ages at which social neurodevelopmental milestones where reached for infants in the birth cohort.

	N Survivors	N Samples	PCR Results
**Samples obtained at delivery**			
Placental swabs	54	56	0 positive
Placental tissue	37	38	0 positive
Cord blood	54	56	0 positive
Maternal blood	53	55	0 positive
**Samples collected longitudinally**			
Vaginal swabs	79	367	0 positive
Breast milk	86	354	2 positive

Table S1: Ebola virus PCR results for specimens collected from birth cohort survivors. Birth specimens were collected across 57 births and 54 survivors, with median (Q1,Q3) [min, max] time in months from EVD symptoms onset to conception of 14 (10, 20) [1, 30]. Twenty specimens were marked "invalid" by the initial Xpert system assay (1 cord blood, 1 maternal blood, 6 vaginal swabs, 12 breast milk) but all 20 subsequently tested negative by the EZ-1 assay. Specimens collected at birth did not always include at least one of each specimen type.

	Adverse outcome	Live birth	Odds Ratio (95% CI) for adverse outcome
Survivors and close contacts	(N=108)	(N=453)	
Mean±SD age at end of pregnancy[1]	28±7	26±7	1.37 (1.08, 1.73)
Mean±SD BMI at PREVAIL III enrollment[1]	25±5	24±5	1.04 (0.83, 1.3)
PREVAIL III enrollment site			
N (%) JFK (ref.)	56 (19)	241 (81)	
N (%) C.H. Rennie	34 (24)	108 (76)	1.35 (0.82, 2.22)
N (%) Duport Road	18 (15)	104 (85)	0.77 (0.43, 1.39)
Education level at PREVAIL III enrollment			
N (%) No formal education (ref.)	15 (13)	100 (87)	
N (%) Primary school or junior high	51 (19)	220 (81)	1.93 (0.95, 3.9)
N (%) Secondary school or above	42 (24)	133 (76)	2.29 (1.16, 4.54)
History of IUFD at PREVAIL III enrollment			
N (%) No (ref.)	81 (19)	357 (82)	
N (%) Yes	27 (22)	96 (78)	0.99 (0.59, 1.66)
PREVAIL III enrollment status			
N (%) survivors	41 (21)	158 (79)	
N (%) close contacts (ref.)	67 (19)	295 (82)	1.13 (0.71, 1.79)
Survivors only	(N=41)	(N=158)	
EVD symptom onset to conception (months)[1]			
Mean±SD	24±15	23±16	1.12 (0.78, 1.61)
Pregnancy end date			
N (%) before 12/24/2015	8 (25)	24 (75)	
N (%) after 12/24/2015 (ref.)	33 (20)	134 (80)	1.44 (0.55, 3.76)

Table S2: Summary of adjusted odds ratios for adverse pregnancy outcome among first reported pregnancies. Adverse outcomes include live birth with subsequent neonatal death, stillbirth (≥20 weeks), spontaneous abortion (<20 weeks) and ectopic pregnancy. Induced abortion was not counted as an adverse outcome. Odds ratios are adjusted for participant age at the time of the outcome, BMI at the time of PREVAIL III enrollment, PREVAIL III enrollment site, education level at the time of PREVAIL III enrollment, and history of IUFD at PREVAIL III enrollment. Odds ratios were estimated using generalized estimating equations (GEE) logistic regression models which accounted for correlatedness arising from repeated measurements (outcomes are reported by fetus, and some pregnancies were multiple pregnancies).

[1] Odds ratio refers to an increase of 1 standard deviation

	Odds Ratio (95% CI) survivor vs. close contact
Live birth	1.04 (0.69, 1.57)
Live birth with subsequent neonatal death	0.42 (0.11, 1.53)
Stillbirth (≥20 weeks)	1.16 (0.37, 3.63)
Spontaneous abortion (<20 weeks)	1.33 (0.78, 2.24)
Induced abortion	0.67 (0.32, 1.43)

Table S3: Adjusted odds ratios for outcomes of first reported pregnancies between seropositive survivors and seronegative close contacts, with missing outcomes multiply imputed. Odds ratios are adjusted for age and PREVAIL III enrollment site.

	Odds Ratio (95% CI) for adverse outcome
Age in years[1]	1.35 (1.07, 1.72)
BMI at PREVAIL III enrollment[1]	1.02 (0.82, 1.28)
PREVAIL III enrollment site	
JFK (ref.)	
C.H. Rennie	1.45 (0.88, 2.41)
Duport Road	0.71 (0.38, 1.31)
Education level at PREVAIL III enrollment	
No formal education (ref.)	
Primary school or junior high	1.72 (0.89, 3.34)
Secondary school or above	2.19 (1.11, 4.33)
History of IUFD at PREVAIL III enrollment	
No (ref.)	
Yes	0.99 (0.6, 1.64)
PREVAIL III enrollment status	
Survivor	1.04 (0.65, 1.66)
Close contact (ref.)	

Table S4: Adjusted odds ratios for adverse pregnancy outcome among first reported pregnancies, with missing outcomes multiply imputed. Adverse outcomes include live birth with subsequent neonatal death, stillbirth (≥20 weeks), spontaneous abortion (<20 weeks) and ectopic pregnancy. Induced abortion was not counted as an adverse outcome. Odds ratios are adjusted for participant age, BMI at the time of PREVAIL III enrollment, PREVAIL III enrollment site, education level at the time of PREVAIL III enrollment, and history of IUFD at PREVAIL III enrollment.

[1] Odds ratio refers to an increase of 1 standard deviation

	Odds Ratio (95% CI) for adverse outcome (analysis A)	Odds Ratio (95% CI) for adverse outcome (analysis B)
Age in years[1]	1.43 (1.14, 1.8)	1.31 (1.06, 1.62)
BMI at PREVAIL III enrollment[1]	1.05 (0.83, 1.31)	1.01 (0.82, 1.23)
PREVAIL III enrollment site		
JFK (ref.)		
C.H. Rennie	1.23 (0.75, 2.02)	1.46 (0.93, 2.29)
Duport Road	0.8 (0.46, 1.41)	0.78 (0.47, 1.3)
Education level at PREVAIL III enrollment		
No formal education (ref.)		
Primary school or junior high	2.22 (1.13, 4.35)	1.28 (0.73, 2.25)
Secondary school or above	2.12 (1.1, 4.09)	1.86 (1.07, 3.22)
History of IUFD at PREVAIL III enrollment		
No (ref.)		
Yes	0.96 (0.59, 1.55)	1.03 (0.64, 1.64)
PREVAIL III enrollment status		
Survivor	1.57 (1.01, 2.43)	0.65 (0.42, 1.01)
Close contact (ref.)		

Table S5: Adjusted odds ratios for adverse pregnancy outcome among first reported pregnancies, with missing outcomes assumed to be adverse outcomes for survivors and live births for contacts in one analysis (A) and the reverse (adverse outcomes for contacts and live births for survivors) in another analysis (B). Adverse outcomes include live birth with subsequent neonatal death, stillbirth (≥20 weeks), spontaneous abortion (<20 weeks) and ectopic pregnancy. Induced abortion was not counted as an adverse outcome. Odds ratios are adjusted for participant age, BMI at the time of PREVAIL III enrollment, PREVAIL III enrollment site, education level at the time of PREVAIL III enrollment, and history of IUFD at PREVAIL III enrollment.

[1]Odds ratio refers to an increase of 1 standard deviation

	Born before 12/24/2015 (N=23)	Born on or after 12/24/2015 (N=82)	Overall (N=105)
Home	5 (21.7)	13 (15.9)	18 (17.1)
Health center	2 (8.7)	7 (8.5)	9 (8.6)
Hospital	9 (39.1)	60 (73.2)	69 (65.7)
Other	7 (30.4)	2 (2.4)	9 (8.6)

Panel A: Birth location for infants enrolled in the birth cohort.

	Pregnancy ended before 12/24/2015 (N outcomes=36)	Pregnancy ended after 12/24/2015 (N outcomes=173)
Live birth	24 (66.7)	134 (77.5)
Live birth with subsequent neonatal death	0 (0)	3 (1.7)
Stillbirth (≥20 weeks)	1 (2.8)	5 (2.9)
Spontaneous abortion (<20 weeks)	7 (19.4)	24 (13.9)
Induced abortion	4 (11.1)	6 (3.5)
Ectopic pregnancy	0 (0)	1 (0.6)
Other[1]	0 (0)	0 (0)

Panel B: Reported pregnancy outcomes among seropositive survivors by pregnancy end date.

[1]One outcome categorized as "Other" was specified as "traumatic abortion < 20 weeks gestation"

Table S6: Birth locations and pregnancy outcomes by pregnancy end date.
